# Biochemical characterization, anti-inflammatory properties and ulcerogenic traits of some cold-pressed oils in experimental animals

**DOI:** 10.1080/13880209.2016.1275705

**Published:** 2017-01-06

**Authors:** Faten M. Ibrahim, Hanan Naeim Attia, Yousreya Aly Aly Maklad, Kawkab A. Ahmed, Mohamed F. Ramadan

**Affiliations:** a Department of Medicinal and Aromatic Plants Research, Pharmaceutical and Drug Industries Division, National Research Centre, Dokki, Giza, Egypt;; b Department of Medicinal and Pharmaceutical Chemistry (Pharmacology group), Pharmaceutical and Drug Industries Research Division, National Research Centre, Dokki, Giza, Egypt;; c Department of Pathology, Faculty of Veterinary Medicine, Cairo University, Giza, Egypt;; d Department of Agricultural Biochemistry, Faculty of Agriculture, Zagazig University, Zagazig, Egypt

**Keywords:** *Syzygium aromaticum*, *Coriandrum sativum*, *Nigella sativa*, LD50, paw oedema, ulcer index

## Abstract

**Context:** Cold-pressed oils (CPO) are commercially available in the market and characterized by their health-promoting properties.

**Objective:** Clove oil (CLO), coriander seed oil (COO) and black cumin oil (BCO) were evaluated for their bioactive lipids. Pharmacological screening was performed to evaluate acute toxicity, anti-inflammatory and ulcerogenic effects as well as histopathological changes in tissues of albino rats fed with CPO.

**Materials and methods*:*** Fatty acids, tocols and total phenolics were analyzed. The acute toxicity test for each CPO was estimated during 14 d. Carrageenan-induced rat paw oedema was used for assessment of anti-inflammatory activity of CPO. Animals were fasted overnight, and *via* oral gavage given indomethacin (10 mg/kg) or CPO (400 mg/kg) to investigate ulcerogenecity. Histopathological changes in liver, kidney, heart, spleen and stomach were screened.

**Results:** Amounts of α-, β-, γ- and δ-tocopherols in CLO were 1495, 58, 4177 and 177 mg/kg oil, respectively. In COO, α, β, γ and δ-tocopherols were 10.0, 18.2, 5.1 and 34.8%, respectively. In BCO, β-tocotrienol was the main constituent. CLO, COO and BCO contained 4.6, 4.2 and 3.6 mg GAE/g, respectively. Acute toxicity test determined that 400 mg/kg of CPO to be used. In the carrageenan model of inflammation, pretreatment of rats with indomethacin (10 mg/kg) or CLO (400 mg/kg) induced a significant (*p* < 0.05) reduction by 31.3 and 27.4%, respectively, in rat paw oedema as compared with the carrageenan-treated group. Indomethacin induced a significant ulcerogenic effect with an ulcer index of 19. Oral treatment of CPO showed no ulcerogenic effect, wherein no histopathological changes were observed.

**Conclusions:** CPO, particularly CLO, could minimize acute inflammation.

## Introduction

Inflammation is one of the most important host defence mechanisms against invading pathogens. Inflammatory reaction is characterized by redness, swelling, heat and pain. However, persistent or over-inflammation leads to tissue damage and possibly the failure of organs (Macarthur et al. 2004). Inflammatory responses are a series of events which depend on the increase in vascular permeability and release of inflammatory mediators, leading to oedema and arrival of leukocytes to inflammation site (Medzhitov 2008).

Natural bioactive compounds, extracts and oils from vegetable, fruits, oilseeds and medicinal plants exhibited strong health-promoting potential that could act against different diseases and exhibit beneficial anti-inflammatory properties. Thus, the demand for natural phytochemicals has greatly increased, and consumers are in quest for natural products for healthy lifestyles (El-Ghorab et al. 2010). Natural products have been evaluated in various animal models to develop new anti-inflammatory agents (Sannigrahi et al. 2010).

Numerous non-traditional oils have been recently introduced to the market without information reported about their composition and functional characteristics. Interest in cold-pressed oils (CPO) has increased, since cold-pressing process involves no heat, no chemical treatments and no refining process. CPO usually contains a high level of bioactive phytochemicals (Ramadan et al. 2012). Recently, it was reported that CPO of clove [*Syzygium aromaticum* L. (Myrtaceae)] oil (CLO), coriander [*Coriandrum sativum* L. (Apiaceae)] oil (COO) and black cumin [*Nigella sativa* L. (Ranunculaceae)] seed oil (BCO) are rich sources of essential fatty acids and bioactive compounds including tocols, phospholipids and phenolic compounds (Ramadan 2013).

Cloves contain a variety of phytochemicals such as sesquiterpenes, tannins and triterpenoids. The main aroma compound in clove buds, eugenol (4-allyl-2-methoxyphenol), was classified by United States Food and Drug Administration (USFDA) to be safe (Miyazawa & Hisama 2003; Gulcin et al. 2012). When administered at levels under 1500 ppm in food, clove oil was listed as a ‘Generally Regarded as Safe’ (GRAS) substance by USFDA. Coriander seeds contain an essential oil rich in linalool (Bajpai et al. 2005; Sreelatha et al. 2009). Coriander-fixed oil contains high levels of petroselinic acid (Δ^6^-*cis*-octadecenoic acid, 18:1*n*-12) as a part of triacylglycerols (Ramadan & Moersel 2002; Ramadan et al. 2008). Thymoquinone and essential oil of black cumin were reported to have different pharmacological properties (Ramadan 2007; Luther et al. 2007; Lutterodt et al. 2010). Black cumin fixed oil is rich in bioactive sterols and tocols (Ramadan & Moersel 2002; Ramadan et al. 2003; Ramadan 2007).

The present study was carried out to (1) determine the fatty acids composition, tocols profile and total phenolics content of CPO, (2) evaluate the acute toxicity of CPO and their anti-inflammatory and ulcerogenic properties and (3) examine histopathologically the liver, heart, kidney, spleen and stomach of rats fed CPO with indomethacin as the reference standard.

## Materials and methods

Female albino rats (age 8 weeks and weight 150–180 g) were obtained from animal breeding house of the National Research Centre (Dokki, Egypt). Rats were housed in clean plastic cages with free access to food (standard pellet diet) and water *ad libitum*, under standardized housing conditions (12 h light/dark cycle, at 23 ± 2 °C, and a minimum relative humidity of 44%) in the laboratory animal room. Animals received human care, according to the criteria outlined in the ‘Guide for the Care and Use of Laboratory Animals’. The Local Ethics Committee at NRC approved the experimental protocols and procedures (Registration no. 90323).

Clove, coriander and black cumin were obtained from a local market (Cairo, Egypt). The plants were taxonomically confirmed as *Syzygium aromaticum*, *Coriandrum sativum* and *Nigella sativa* by Dr. A. Salma at the herbarium of Department of Botany, Zagazig University (Egypt). A voucher specimen numbers 1523, 1597 and 1720 were used wherein the specimen was lodged at Department of Botany, Zagazig University (Egypt). Oils were cold-pressed by local extractor (Cairo, Egypt). Indomethacin, Tween 80 and carrageenan were obtained from Sigma (St. Louis, MO). All chemicals and reagents used were of analytical grade.

### Gas chromatography (GC) of fatty acids methyl esters (FAME) in CPO

Fatty acids in CPO were transesterified to FAME by *N*-trimethylsulfoniumhydroxide (Macherey-Nagel, Germany) according to Arens et al. (1994). Shimadzu GC-14A equipped with flame ionization detector (FID) and C-R4AX chromatopac integrator (Kyoto, Japan) was used. The flow rate of the carrier gas helium was 0.6 mL/min and the split value with a ratio of 1:40. A sample of 1 μL was injected on a 30 m × 0.25 mm × 0.2 μm film thickness Supelco SPTM-2380 (Bellefonte, PA) capillary column. The injector and the FID temperature was set at 250 °C. The initial column temperature was 100 °C programmed by 5 °C/min until 175 °C and kept 10 min at 175 °C, then 8 °C/min until 220 °C and kept 10 min at 220 °C. A comparison between the retention times of the samples with those of an authentic standard mixture (Sigma, St. Louis, MO; 99% purity specific for GC), run on the same column under the same conditions, was made to facilitate identification.

### High performance liquid chromatography (HPLC) of tocols in CPO

A solution of 250 mg of CPO in 25 mL *n*-heptane was directly used for the HPLC (Ramadan 2013). The HPLC analysis was conducted using a Merck Hitachi low-pressure gradient system (Merck, Darmstadt, Germany), fitted with an L-6000 pump, a Merck-Hitachi F-1000 Fluorescence Spectrophotometer (Merck, Darmstadt, Germany) (the detector wavelength was set at 295 nm for excitation and at 330 nm for emission) and a D-2500 integration system; 20 μL of the samples were injected by a Merck 655-A40 Autosampler onto a Diol phase HPLC column 25 cm 94.6 mm ID (Merck, Darmstadt, Germany) using a flow rate of 1.3 mL/min. The mobile phase used was *n*-heptane/*tert*-butyl methyl ether (99:1, v/v).

### Extraction and quantification of phenolic compounds in CPO

CPO (1 g) was dissolved in *n*-hexane (5 mL) and mixed with 10 mL methanol–water (80:20, v/v) in a glass tube for 2 min in a vortex (Ramadan et al. 2010). After centrifugation at 3000 rpm for 10 min, the hydroalcoholic extracts were separated from the lipid phase by using a Pasteur pipette then combined and concentrated *in vacuo* at 30 °C until a syrup consistency was reached. The lipidic residue was redissolved in 10 mL methanol:water (80:20, v/v) and the extraction was repeated twice. Hydroalcoholic extracts were redissolved in acetonitrile (15 mL) and the mixture was washed three times with *n*-hexane (15 mL each). Purified phenols in acetonitrile were concentrated *in vacuo* at 30 °C then dissolved in methanol for further analysis. Aliquots of phenolic extracts were evaporated to dryness under nitrogen. The residue was redissolved in 0.2 mL water and diluted (1:30) Folin–Ciocalteu’s phenol reagent (1 mL) was added. After 3 min, 7.5% sodium carbonate (0.8 mL) was added. After 30 min, the absorbance was measured at 765 nm using a UV-260 visible recording spectrophotometer (Shimadzu, Kyoto, Japan). Gallic acid was used for the calibration and the results of triplicate analyses are expressed as parts per million of gallic acid.

### Pharmacological properties of CPO

#### Acute toxicity

The acute toxicity test for CPO was estimated to evaluate any possible toxicity. The test was performed according to Economic Cooperation and Development (OECD) 423 guidelines (OECD 2001). Five female albino rats (*n* = 5) were fasted overnight with free access to drinking water then given BCO, COO and CLO at graded doses up to 4000 mg/kg. The dosing patron was 500, 1000, 1500, 2000, 2500, 3000, 3500 and 4000 mg/kg body weight for BCO, COO and CLO oils, while the control group received only the normal saline. Animals were closely monitored for 24 h and daily for 14 d until early signs of toxicity and/or mortality were observed. Death of half of examined animals was observed at 4000 mg/kg. Therefore, 400 mg/kg (1/10 of 4000 mg/kg) was selected as the maximum safety dose with descending dose levels.

#### Anti-inflammatory activity

Carrageenan-induced rat paw oedema has been used for assessment of the anti-inflammatory activity of CPO, according to Ibrahim et al. (2011). Female albino rats were acclimatized for 7 d before treatment and randomly assigned into six groups of eight rats each. Dosages of indomethacin and CPO were freshly prepared in 7% Tween 80 saline solution (0.9% w/v) and given *via* oral gavage. Animals were fasted overnight, assigned into groups and orally given indomethacin (10 mg/kg) according to Nkeh-Chungag et al. (2009) as well as CPO (400 mg/kg). One hour following the treatments, paw swelling was induced by subcutaneous injection of 100 μL of 1% sterile lambda carrageenan suspension in saline into the plantar region of the right hind paw of all groups (Juhas et al., 2009). A control untreated group received 100 μ of carrageenan 1% (w/v). An equivalent volume of saline was injected intra-plantarly in the right hind paw of another group of animals which served as normal group. Volume of the paw oedema was measured in each animal using a plethysmometer (plethysmometer 7150, Ugo Basile, Monvalle, Italy) with a precision of two decimal places. The volume of paw oedema was determined immediately before (baseline volume as 100%) and 4 h after carrageenan administration (Ibrahim et al. 2011) then expressed as percentage of the basal volume (before injection) (Salter-Cid et al. 2005).

#### Ulcerogenic effect

Ulcerogenecity was investigated by oral administration of 10 mg/kg indomethacin or CPO to overnight-fasted animals. After 4 h, rats were euthanized and the stomach was removed and opened along the greater curvature, rinsed with saline and examined by a magnifier lens (10×) to assess the presence of lesions and formation of ulcers. The numbers of ulcers were counted and the severity was determined by giving scores between 0 and 4. Ulcer index is defined as the sum of the average ulcer number, average severity of ulcers and the percentage incidence of ulcer divided by 10 (Dashputre & Naikwade 2011).

#### Histopathological examination

After the previous treatments, the animals were sacrificed wherein liver, kidney, heart, spleen and stomach were removed, washed with normal saline, fixed in 10% formalin, dehydrated in ascending grades of alcohol and embedded in paraffin wax. Paraffin sections were taken at 5 μm thick and stained with haematoxylin & eosin (H&E). The sections were examined for histopathological changes (×160) under light microscope. The liver, kidney, heart, spleen and stomach fields were scored according to Michael (2008).

### Statistical analysis

Statistical analysis was done using Graphpad Prism (version 5, Graphpad Inc., San Diego, CA), and the values were expressed as mean ± SEM. The statistical significance of differences between the means was analyzed using one-way analysis of variance (ANOVA) followed by Tukey’s test for comparison between different treatment groups. Statistical significance was set at *p* ≤ 0.05.

## Results

### Fatty acids composition of CPO

**Figure 1.**
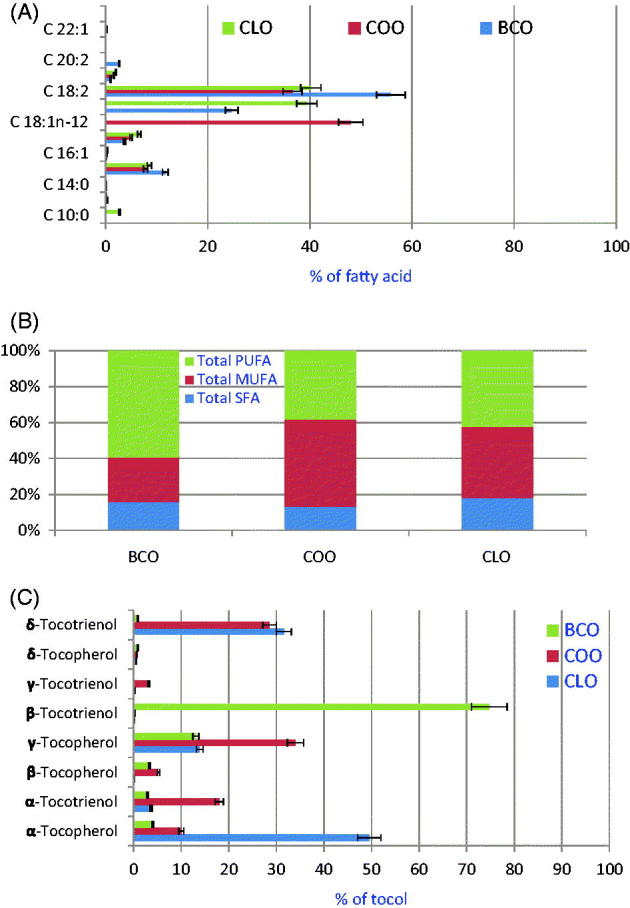
Relative percentages of fatty acids (A) levels of SFA, MUFA and PUFA (B), and levels of tocols (C) in CPO.

Figure 1(A) presents the relative percentage of fatty acids in CPO. In CLO, linoleic and oleic acids were the main unsaturated fatty acids accounting for almost 80% of total identified FAME, wherein palmitic and stearic acids were the major saturated fatty acids (SFA), accounting for almost 15% of total fatty acids. The levels of C16:0, C18:0, C18:1*n*-9, C18:2 and C18:3 were 8.5, 6.6, 39, 40 and 1.9%, respectively. CLO contained high levels of monounsaturated fatty acids (MUFA, 39.7% of total fatty acids) and polyunsaturated fatty acids (PUFA, 42.0% of total fatty acids). A striking feature of COO was the high level of MUFA. In COO, petroselinic acid (C18:1*n*-12) was the main fatty acid accounted for 48%, followed by linoleic acid (36.7%). The amount of petroselinic acid detected was similar to that reported by Ramadan and Moersel (2002) and Ramadan et al. (2008). Levels of C16:0, C18:0, and C18:3 in COO were 7.8, 4.9 and 1.6%, respectively. A striking feature of BCO was the high amount of unsaturated fatty acids (PUFA and MUFA). Linoleic acid (18:2*n*-6, 55%), oleic acid (18:1*n*-9, 24%) and palmitic acid (16:0, 15%) were the main fatty acids in BCO.

Figure 1(B) presents the levels of SFA, MUFA and PUFA in CPO. CLO and BCO were characterized by high amounts of PUFA, while COO was characterized by high amounts of MUFA.

### Tocols and phenolic compounds profile of CPO

Figure 1(C) presents the percentages of tocols in CPO under study. In general, CPO contained high amounts of unsaponifiables. In CLO, COO and BCO, the levels of unsaponifiables were 2.53, 3.94 and 1.79 g/kg oil, respectively. In CLO, the amounts of α-, β-, γ- and δ-tocopherols were 1495, 58, 4177 and 177 mg/kg oil, respectively. Furthermore, the levels of α-, β-, γ- and δ-tocotrienols were 1113, 52, 86 and 9481 mg/kg oil, respectively. In CLO, about half of total tocols amount was as α-tocopherol followed by δ-tocotrienol (*ca*. 32%) and γ-tocopherol (*ca*. 14%). In COO, the percentages of α-, β-, γ- and δ-tocopherols were 10.0, 18.2, 5.1 and 34.8%, respectively. In BCO, β-tocotrienol (1190 mg/100 g oil) was the main constituent followed by γ-tocopherol (208 mg/100 g oil). Other tocols were measured in lower amounts. CPO were characterized by high amounts of total phenolic compounds. In our study, CLO, COO and BCO contained 4.6, 4.2 and 3.6 mg GAE/g, respectively.

### Anti-inflammatory activity

Data presented in Table 1 revealed that the subplantar injection of carrageenan (100 μL, 1%) exerted a significant 1.5-fold increase in the hind paw volume (4 h post-treatment) in comparison with normal rats. Pretreatment of rats with indomethacin (10 mg/kg) or CLO (400 mg/kg) induced a significant (*p* < 0.05) reduction by 31.3 and 27.4%, respectively, in rat paw oedema as compared with the carrageenan-treated group. On the contrary, administration of BCO or COO at a dose of 400 mg/kg showed an insignificant reduction in paw oedema as compared with the carrageenan-treated animals and was significantly (*p* < 0.05) higher than that of the normal and indomethacin treated groups.

**Table 1.** Effect of oral administration of indomethacin and CPO on paw volume and ulcerogenicity (4 h post-injection of carrageenan-induced rat paw edema) in the adult female albino rats.

	Paw volume	Ulcerogenic effect					
Group and doses	% Baseline value	Number of animals	Number of animals showing ulcer	Mean No of ulcer	Severity of ulcer	% incidence of ulcer/10	Ulcer index
Normal (100 μL Saline)	100	8	0	0	0	0	0
Carrageenan (100 μL, 1%)	147.6*^b^ ± 2.56	0	0	0	0	0	0
Indomethacin (10 mg/kg PO)	115.7^a^ ± 4.14	8	6	6.9 ± 0.7	2.5 ± 0.33	10	19.4*
BCO (400 mg/kg PO)	137.8*^b^ ± 8.21	8	0	0	0	0	0
COO (400 mg/kg PO)	138.8*^b^ ± 4.79	8	0	0	0	0	0
CLO (400 mg/kg PO)	120.2^a^ ± 0.92	8	0	0	0	0	0

Data are shown as mean ± S.E.M. (*n =* 8).

* Significantly different from normal value (*p* < 0.05).

a Significantly different from carrageenan-treated group (*p* < 0.05).

b Significantly different from indomethacin-treated group (*p* < 0.05).

### Ulcerogenicity

Table 1 revealed that oral administration of indomthacin (10 mg/kg) induced a significant ulcerogenic effect with respect to the normal animals at *p* < 0.05, where its ulcer index was 19. Meanwhile, oral treatment of COO, BCO and CLO lacked an ulcerogenic effect in comparison with the normal group. However, COO showed minimal haemorrhage.

### Histopathological properties

**Figure 2.**
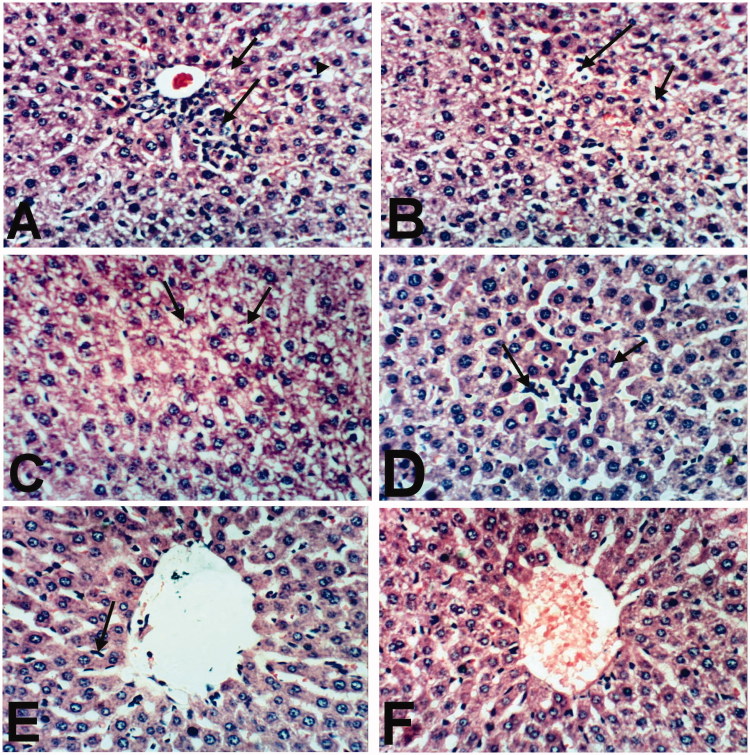
Liver of rat treated with (A) indomethacin showing cytoplasmic vacuolation of hepatocytes (small arrow), small focal hepatic necrosis associated with inflammatory cells infiltration (large arrow) and Kupffer cells activation (arrow head). (B) Indomethacin showing cytoplasmic vacuolation of hepatocytes (small arrow) and sinusoidal leukocytosis (large arrow). (C) BCO showing cytoplasmic vacuolization of hepatocytes (arrows) and Kupffer cells activation (large arrow). (D) COO showing Kupffer cells activation (small arrow) and sinusoidal leukocytosis (large arrow). (E) CLO showing slight activation of Kupffer cells (arrow). (F) Control showing the normal histological structure of hepatic lobule. (H & E x 400).

Liver of rats treated with indomethacin revealed cytoplasmic vacuolation of hepatocytes (Figure 2(A) and (B)), small focal hepatic necrosis associated with inflammatory cells infiltration (Figure 2(A)) and Kupffer cells activation (Figure 2(A) and (B)). Moreover, liver of rat treated with BCO showed Kupffer cells’ activation and cytoplasmic vacuolization of hepatocytes (Figure 2(C)). Examined sections from rat treated with COO showed Kupffer cells activation and sinusoidal leukocytosis (Figure 2(D)). Meanwhile, an improvement in the histopathological picture was noticed in liver of rat treated with CLO, as the examined sections revealed only slight activation of Kupffer cells (Figure 2(E)). However, liver of control, untreated rat, revealed the normal histological structure of hepatic lobule (Figure 2(F)).

**Figure 3.**
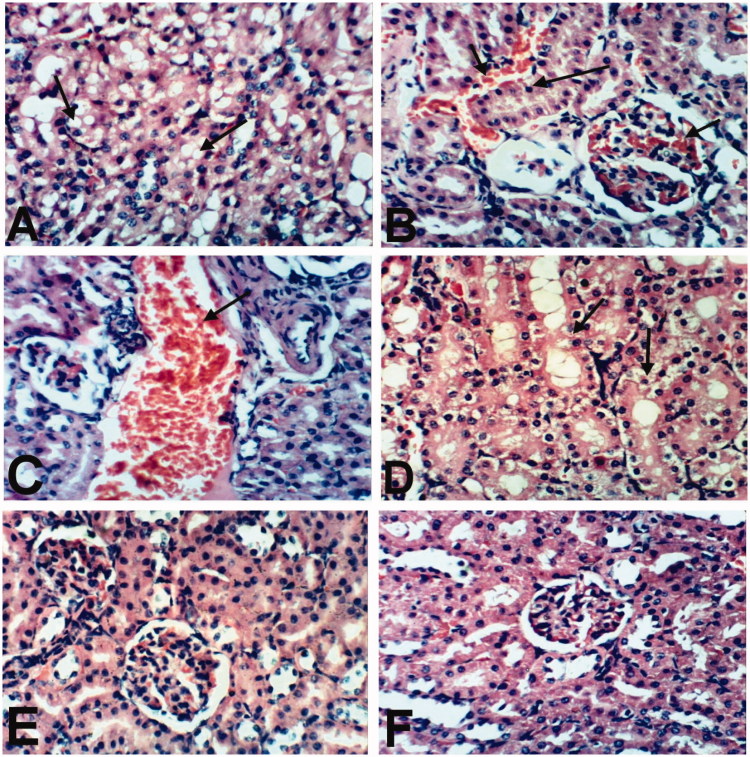
Kidney of rat treated with (A) indomethacin showing vacuolization of epithelial lining renal tubules (arrow). (B) Indomethacin showing congestion of intertubular blood vessels and glomerular tuft (small arrow) and pyknosis of the nuclei of renal tubular epithelium (large arrow). (C) BCO showing dilatation and congestion of renal blood vessel (arrow). (D) COO showing vacuolation of epithelial lining renal tubules (arrows). (E) CLO showing no histopathological changes. (F) Control showing the normal histological structure of renal parenchyma. (H & E x 400).

Examination of kidneys of rats treated with indomethacin revealed vacuolization of epithelial lining renal tubules (Figure 3(A)), congestion of intertubular blood vessels and glomerular tuft as well as pyknosis of the nuclei of renal tubular epithelium (Figure 3(B)). Meanwhile, kidneys of rats treated with BCO showed mild histopathological changes. Some examined sections from this group showed dilatation and congestion of renal blood vessel (Figure 3(C)), whereas other sections from this treatment revealed no histopathological changes. Kidneys of rats treated with COO showed no changes except vacuolation of epithelial lining renal tubules (Figure 3(D)). An improved histopathological picture was noticed in kidneys of rat treated with CLO as the examined sections revealed no histopathological changes (Figure 3(E)). Moreover, kidneys of untreated rats showed the normal histological structure of renal parenchyma (Figure 3(F)).

**Figure 4.**
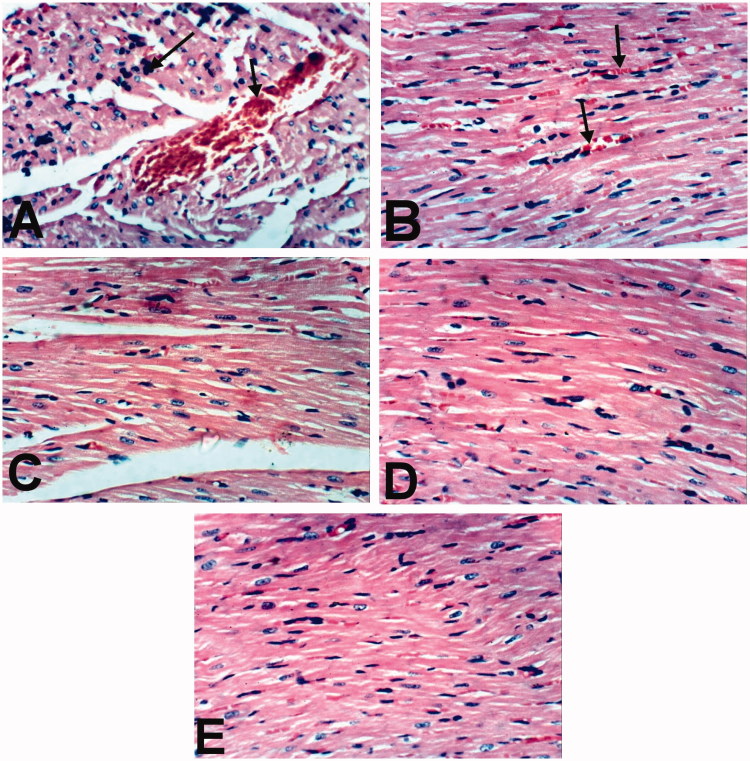
Heart of rat treated with (A) Indomethacin showing congestion of myocardial blood vessel (small arrow) and few mononuclear inflammatory cells infiltration (large arrow). (B) BCO showing slight congestion of myocardial blood capillaries (arrow). COO (C), CLO (D) and control (E) showing no histopathological changes (H & E x 400).

Heart of rats treated with indomethacin showed congestion of myocardial blood vessel and few mononuclear inflammatory cells infiltration (Figure 4(A)). However, slight congestion of myocardial blood capillaries was the only histopathological finding observed in heart of rats treated with BCO (Figure 4(B)). Moreover, heart of rats treated with COO, CLO and control revealed no histopathological changes (Figure 4(C,D) and (E)).

**Figure 5.**
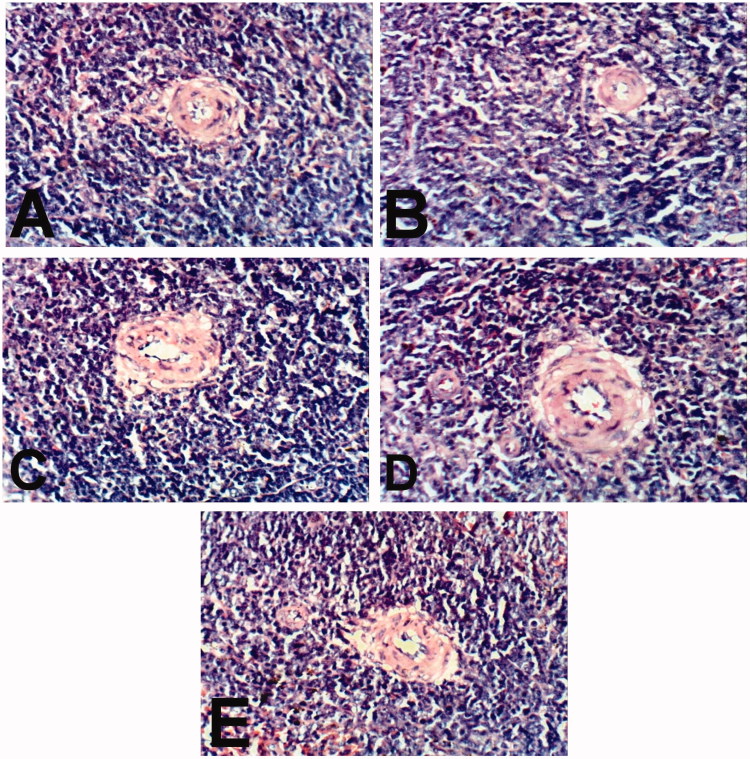
Spleen of rats treated with indomethacin (A), BCO (B), COO (C), CLO (D), and control (E) showing no histopathological changes (H & E x 400).

**Figure 6.**
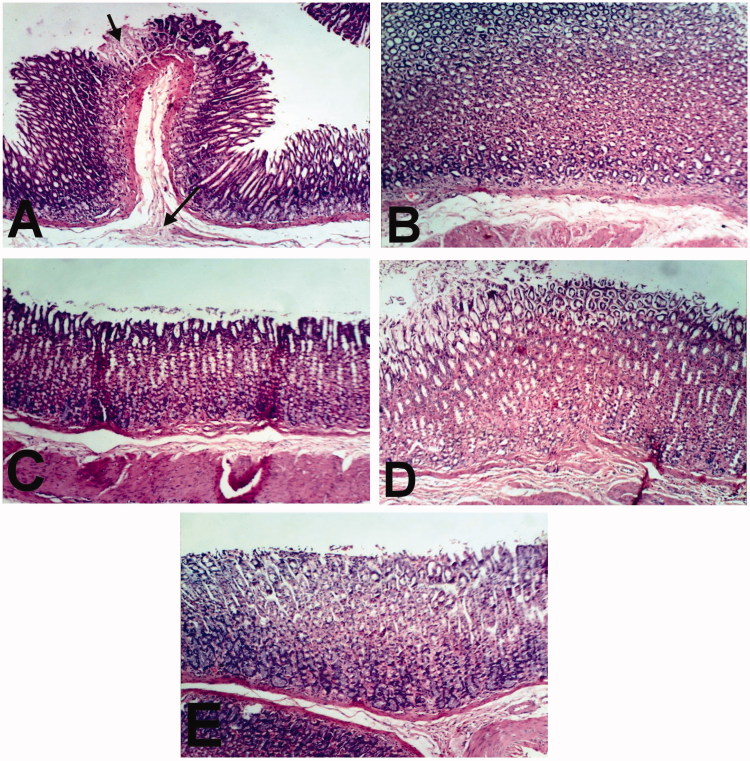
Stomach of rat treated with (A) indomethacin showing focal necrosis of gastric mucosa (small arrow) and submucosal edema (large arrow). BCO (B), COO (C), CLO (D), and control (E) control showing no histopathological changes (H & E x 100).

Spleen of rat treated with indomethacin and CPO as well as normal control showed no histopathological changes (Figure 5). Examination of stomach of rat treated with indomethacin revealed congestion of mucosal and submucosal blood vessels, focal necrosis of gastric mucosa and submucosal edema (Figure 6(A)). However, stomach of rats treated with CPO and control showed no histopathological changes (Figure 6(B,C,D) and (E)).

## Discussion

Inflammation plays a major role in most chronic illnesses, including cardiovascular, metabolic, pulmonary and neoplastic diseases. Health-promoting effects of edible oils can be attributed to its specific fatty acid profile (levels of MUFA and PUFA as well as *omega*-6PUFA/*omega*-3PUFA ratio) and its richness in bioactive phytochemicals including phenolics, phytosterols, tocols and carotenoides. CPO under study was characterized by relative high levels of PUFA and MUFA which makes them a special constituent for functional applications. The benefits of PUFA and MUFA, in inflammation, diabetes, cardiovascular and other diseases were reported. MUFA were shown to lower LDL (low-density lipoproteins) cholesterol and retain HDL (high-density lipoproteins) cholesterol. In addition, PUFA reduce both LDL and HDL cholesterol levels in human blood (Ramadan et al. 2010).

The consumption of omega-3 PUFA plays a protective role in inflammatory bowel disease (Calder 2008; Lachs et al. 2009). Omega-3 PUFA exhibited anti-inflammatory traits due to the activity of eicosanoids derived from eicosapentaenoic acid (20:5n-3) that can be synthesized after ingestion from α-linolenic acid (18:3 *n*-3) (Reifen et al. 2015). Omega-3 PUFA benefit brain health by modulating neuroimmune and apoptotic pathways, changing membrane function and/or competing with *n*‐6 PUFAs, the precursors of inflammatory mediators (Song et al. 2016). Omega-3 PUFA reduced mucosal damage in rats with experimental colitis (Gil 2002), as assessed by biochemical and histological markers of inflammation. The results of the present study are in agreement with Reifen et al. (2015) who reported that colitic rats fed sage oil diets had a lower inflammatory response, improved histological repair and had less necrotic damage in the mucosa when compared to the corn and fish oil groups. Colonic damage and myeloperoxidase activity were significantly lower. Gastric lesion induction is affected by endogenous gastroprotective substance, oxidative stress and inflammation. *Hippophae rhamnoides* pulp and seeds oils rich in MUFA and PUFA showed dose-dependent anti-ulcerogenic properties against ulcerogenesis (Suleymanov et al. 2004). The selected CPO were devoid of ulcerogenic activity at the tested dose used in the study.

Tocols are the major lipid-soluble, membrane-localized antioxidants in humans. CPO under study contained high amounts of tocols. α- and γ-Tocopherols proved to be the major tocopherols in vegetable oils and fats. Müller et al. (2010) showed that the reducing ability and radical chain-breaking activity of the several vitamin E forms depends on the circumstances under which the assays are performed. Tocopherols are the most efficient antioxidants, while β-tocopherol has 25–50% of the antioxidative activity of α-tocopherol and γ-isomer 10–35% (Ramadan 2013). Vitamin E impacts on disorders were extensively reported in humans or experimental animals. Treatment with tocols may afford beneficial effects in attenuating the formation of the gastric lesions. α-Tocopherol and tocotrienol reduced gastric lesion index in experimental animals exposed to ulcerogens (Cuevas et al. 2011; Fahami et al. 2012: Kamisah et al. 2014). Administration of α-tocopherol and tocotrienol was able to reduce the increase in oxidative stress in these models of gastric lesions (Cuevas et al. 2011; Ishihara et al. 2008; Kamisah et al. 2011). α-Tocotrienol showed lower gastric lesion index than α-tocopherol in rats exposed to repetitive restraint stress (Kamisah et al. 2014).

Cold-pressed BCO, COO and CLO contained high amounts of TPC. Phenolic compounds in edible oils (i.e., olive oil) exhibited a broad spectrum of functional traits, including antiradical, antioxidant and anti-inflammatory impacts (Cárdeno et al. 2013; Sánchez-Fidalgo et al. 2012). Rosillo et al. (2014) reported the anti-inflammatory and protective impacts of the phenolics extract from olive oil in a collagen-induced arthritis model.

These previous studies are in accordance with our present investigation, particularly for CLO, which revealed an improvement in the carrageenan model of inflammation. Noteworthy, CLO anti-inflammatory effect was comparable with indomethacin with the advantage of having no ulcerogenic activity.

## Conclusions

CPO rich in bioactive compounds appear to be promising oils for safe use in pharmaceutical products and folk medicine. In conclusion, CPO under study possesses significant anti-inflammatory activity and no ulcerogenic effect, which may be attributed to the presence of tocols, and other bioactive phenolic compounds. After pharmacological and toxicological screening, CPO under study could be used as anti-inflammatory agents. The pharmacological traits and the lack of toxicity render CPO a valuable candidate for further studies as an agent for inflammation treatment. Interestingly, CLO could be considered superior to both BCO and COO with respect to anti-inflammatory and gastric safety profile at the tested dose in this investigation.
